# Enzymatic production of bioactive peptides from scotta, an exhausted by-product of ricotta cheese processing

**DOI:** 10.1371/journal.pone.0226834

**Published:** 2019-12-30

**Authors:** Stefania Monari, Maura Ferri, Claudio Russo, Barbara Prandi, Tullia Tedeschi, Paolo Bellucci, Angelo Vittorio Zambrini, Emanuela Donati, Annalisa Tassoni

**Affiliations:** 1 Department of Biological, Geological, Environmental Science, University of Bologna, Bologna, Italy; 2 Department of Civil, Chemical, Environmental, and Materials Engineering, University of Bologna, Bologna, Italy; 3 Territorial and Production Systems Sustainability Department, Biotechnologies and Agroindustry Division, BioProducts and BioProcesses, ENEA Italian National Agency for New Technologies, Energy and Sustainable Economic Development, Roma, Italy; 4 Department of Food and Drug, University of Parma, Parma, Italy; 5 Department of Quality, Innovation, Safety, Environment, Granarolo S.p.A., Bologna, Italy; Technical Educational Institute of Peloponnese, GREECE

## Abstract

The present work reports the enzymatic valorisation of the protein fraction of *scotta*, a dairy by-product representing the exhausted liquid residue of ricotta production. Scotta was subjected to ultra-filtration with membrane cut-offs from 500 to 4 kDa and the obtained protein-enriched fractions were used for the optimization of enzyme-based digestions aimed at producing potentially bioactive peptides. Nine different commercial proteases were tested and the best digestion conditions were selected based on protein yield, fraction bioactivity and foreseen scale up processing costs. Scale up of the 3% Pancreatin or 5% Papain processes was performed up to 2 L (37°C or 60°C respectively, 1 h incubation), and the digestion efficiency increased with the reaction volume as well as antioxidant activity (up to 60 gBSA eq/L and to 1.7 gAA eq/L). Retentate 1 digested fractions also showed, for the first time in dairy-based peptides, anti-tyrosinase activity, up to 0.14 gKA eq/L. Digested proteins were sub-fractionated by means of physical membrane separations and 30–10 kDa fraction from Papain treatment showed the highest antioxidant and anti-tyrosinase activities. The peptide sequence of the most bioactive fractions was achieved.

## Introduction

Food industry produces a considerable amount of by-products and losses resulting both from animal and vegetable processing during the whole food production chain. The disposal of these residues, that might have an important environmental impact, requires high economic and energetic costs, so the search of new solutions for their reuse and recycle is increasingly required. In particular, by-product exploitation can be achieved by extracting high-value components, such as proteins and peptides, which can be used for food, feed, cosmetic and nutraceutical purposes.

Milk is one of the most abundant agricultural products in the Mediterranean area, and dairy milk factories generate high amounts of by-products and wastes. Whey is the main dairy by-product resulting from cheese manufacturing and corresponds to the liquid fraction left after milk curdling and straining. A minor percentage of whey is employed to produce ricotta cheese, which is obtained after whey heating at 85–90°C for 20–30 minutes to allow proteins precipitation. The exhausted whey liquid fraction, after ricotta production, is called *scotta* and represents the most abundant cheese-making residue produced in southern Europe, particularly in Italy. In fact, ricotta is a typical Italian dairy product even though whey cheeses are manufactured all over the world and, therefore, the disposal of their making residues is an issue present in many countries [1, 2]. Scotta from bovine whey is composed by proteins (0.15–0.22%), salts (1–1.13%), lactose (3.7–5.0%) and fats (0.1–0.3%) and it is characterized by high BOD (Biochemical Oxygen Demand) and COD (Chemical Oxygen Demand) values, 50 g/L and 80 g/L respectively [2, 3]. Milk proteins are composed by two main groups: caseins, which represent their major amount (about 80%), and whey proteins, such as β-lactoglobulin, α-lactalbumin, serum albumin and immunoglobulins [4].

Treatment of dairy wastes to reduce their polluting impact represents one of the biggest problems for companies in the sector. In fact, because of the high content of perishable organic matter, whey and scotta cannot be disposed directly into water bodies, and their treatment by using conventional biological purification systems is considerably difficult and costly. Several industrial solutions were developed for the exploitation of whey dairy waste including biogas and ethanol production by fermentation [5], as food and feed ingredients [6] and as feedstocks for biopolymer production [7]. On the contrary, a lower amount of studies were performed on scotta, mainly dealing with bio-ethanol production [3, 8], bioconversion of lactose into lactic acid [9] and obtainment of fermented drinks by lactic acid bacteria (LAB) [10].

In recent years, many efforts were devoted to the valorisation of industrial food processing residual proteins in particular as food and feed ingredients [11, 12]. Proteolytic enzyme modification is an efficient approach to improve the functional properties of animal and plant food proteins [13, 14] by generating potentially bioactive peptides. The greatest advantages of using enzymes during industrial food processing are their environmental friendliness and their high consumer acceptance as they are generally perceived as natural components. Bioactive peptides usually consist of chains of 2–20 amino acids that, in addition to their nutritional role, also exert several biological activities with potentially beneficial effects on human health [11, 12, 15]. Many of the food-derived peptides demonstrated in fact anti-hypertensive, anti-inflammatory, antimicrobial, antithrombotic, antidiabetic and antioxidant properties [14–17]. In particular antioxidant capacity of bioactive peptides derived from animal (i.e. fish, milk and dairy products) and plant (i.e. sweet potato and soybean) proteins, is well known and this property is often considered a significant indicator of good product quality [18, 19]. Generally, milk proteins are one of the most important sources of bioactive peptides showing several multifunctional properties and physiological functions [20–22]. Nowadays several peptides derived from milk and whey proteins are used as additive in food and feed industries, both for their antioxidant and texture-modifying properties [23], conversely, no specific research on the industrial valorisation of scotta proteins and peptides, was previously published.

Increasing importance is also gaining the potential anti-tyrosinase activity of new extracts. This capacity was usually ascribed to plant secondary metabolites [24–27], but was also reported for proteins and peptides coming from animal [28] and plant [14] sources. Tyrosinase inhibitors are valuable in nutraceutical and functional food sectors as browning preventing [26] or as anti-aging agents [24].

In order to valorise the dairy scotta industrial by-product from ricotta cheese production, the present work aimed at the optimization of enzyme-based digestion methodologies to obtain potentially bioactive peptides showing biological activities. These active peptides could find their application as ingredients in the nutraceutical, food and cosmetics fields. In addition, being ricotta and other whey cheeses (and consequently their residues) produced in large volumes all over the world, finding a way to reuse scotta would allow to reduce environmental impacts and production costs and therefore to alleviate the economic burden for cheese producers.

## Materials and methods

### Materials

Bovine scotta (S) samples, produced at Granarolo industrial site of Usmate or Capurso dairy of Gioia del Colle by using cow milk, were provided by Granarolo S.p.A. (Bologna, Italy) between 2014 and 2017. Chemical-physical characterisation of the same type of scotta was published by Monti et al. [2] and, in particular total nitrogen content was on average 61 mg/100g and fat content was 160 mg/100g.

No pre-treatment (such as fat removal) was applied to scotta which was directly subjected to two successive ultrafiltration steps by means of different molecular weight cut-off membranes in collaboration with ENEA (Rome, Italy) at the Granarolo industrial site of Gioia del Colle. Experimental filtration tests with membrane technologies were carried out with pilot plants operating in batch conditions, collecting the permeate and recirculating the retentate inside the feed tank until the desired volumetric concentration ratio was reached. For all the tests polymeric spiral-wound membranes 4040 were used.

In the first four tests, scotta was treated with two successive filtration stages, with the permeate of the first stage re-loaded in the next stage; in the fifth test, a single filtration step was carried out (Table 1).

**Table 1:** Main features of analysed different scotta batches and cut-off of performed ultrafiltration steps. R1, retentate 1; R2, retentate 2.

Batch	Harvest season	Production site	Membranes and manufacturers	Membrane cut-off (kDa)	
				R1	R2
**1**	December, 2014	Usmate	R1: EW4040F1020—GE Water & Process Technologies	100	30
			R2: ISUH0304040C1—Microdyn-Nadir		
**2**	July, 2015	Gioia del Colle	R1: EW4040F1020 –GE Water & Process Technologies	100	30
			R2: ISUH0304040C1—Microdyn-Nadir		
**3**	December, 2015	Gioia del Colle	R1: ISUC5004040C1—Microdyn-Nadir	500	30
			R2: ISUH0304040C1—Microdyn-Nadir		
**4**	May, 2016	Gioia del Colle	R1: ISUC5004040C1—Microdyn-Nadir	500	4
			R2: ISUH0044040C1—Microdyn-Nadir		
**5**	May, 2017	Usmate	R1: PW4040C30—GE Water & Process Technologie	10	-

Four scotta batches (batch 1 to 4) were treated, different in source and harvest season, and, after different ultrafiltration processes, from each batch two different samples were generated: retentate 1 (R1) and retentate 2 (R2) (Table 1); in batch 5 only R1 sample was generated (Table 1). All the samples were stored at -20°C until further analysis.

The dry weight (gDW) was calculated by weighing 1 mL of each sample (triplicate biological repetitions) after drying at 80°C for 2 days (Table 2).

**Table 2:** Total protein and dry weight contents of scotta (S), retentate 1 (R1) and retentate 2 (R2) fractions from each processed initial feedstock batch.

Batch N°	S		R1		R2	
	Total proteins (gBSA eq/L)	Dry weight (g/L)	Total proteins (gBSA eq/L)	Dry weight (g/L)	Total proteins (gBSA eq/L)	Dry weight (g/L)
**1**	6.5 ± 1.7	54.4 ± 8.1	89.0 ± 5.6	137.8 ± 9.3	2.8 ± 0.9	57.4 ± 9.7
**2**	3.1 ± 0.3	62.9 ± 0.9	37.0 ± 0.2	126.5 ± 0.8	1.5 ± 0.1	66.3 ± 0.3
**3**	2.9 ± 0.2	44.7 ± 7.2	29.3 ± 1.0	82.6 ± 0.5	13.7 ± 1.4	67.9 ± 0.4
**4**	2.3 ± 0.3	50.0 ± 8.7	24.0 ± 4.6	87.1 ± 1.5	9.4 ± 1.0	138.5 ± 2.0
**5**	2.0 ± 0.6	43.5 ± 7.0	63.4 ± 0.5	119.4 ± 6.6	-	-

### Protease treatments and scale up

Nine different proteases were tested during initial digestion trials (20 mL final volume) on batch 1 at 1-5-10% (w/w, g enzyme/gDW) enzyme/substrate ratio (E/S), 2 h of incubation, 140 rpm stirring, pH 7.0 and at the following incubation temperature according to the manufacturer’s instructions: Neutrase 0.8L (50°C), Flavourzyme 500L (50°C), Alcalase 2.4L (60°C) and Protamex (60°C) purchased from Novozymes A/S (Denmark), and Papain (60°C), Pancreatin (37°C), Bromelain (37°C), Chymotrypsin (37°C) and Trypsin (37°C) purchased from Sigma-Aldrich (Milan, Italy). Not digested (ND) control samples were performed at the same incubation conditions but without the addition of the enzymes. The digestions were stopped by boiling the samples in a water bath for 10 min to inactivate the enzymes and the samples were stored at -20°C. For the treatment of batch 2, 3, 4 samples, the four best performing enzymes and digestion conditions for each type of sample (S, R1 and R2), were selected, and E/S ratio and time of incubation were further optimised.

Successively, ten-fold scale up experiments were performed in 500 mL flasks containing 200 mL of final volume reaction, by using selected E/S ratios, 1 h incubation at 140 rpm stirring, at previous digestion conditions depending on the protease. One hundred-fold scale up digestions were performed by means of a bioreactor (Applikon biotechnology, Delft, Netherlands) in a 2.5 L glass vessel containing 2 L of final volume reaction (R1, batch 5), 3% Pancreatin or 5% Papain E/S ratio, 37°C or 60°C respectively, 1 h incubation at 350 rpm stirring.

At least two replicates were performed for all digestion conditions. Samples were stored at -20°C until further analysis.

### Quantification and molecular mass characterization of proteins

Total protein content was determined using the method described by Lowry [29]. The results were expressed as gram of bovine serum albumin (BSA) equivalents per litre of sample (gBSA eq/L) by means of a dose-response calibration curve (between 0 and 200 μg of BSA).

The molecular mass distribution of initial protein fractions and of digestates was analysed by mono-dimensional SDS-PAGE with 16% w/v acrylamide [30] (see an example in S1 Fig).

### Fractionation process

The 2 L digestates were sequentially fractioned by means of centrifugation columns (Vivaspin 20, Ultrafiltration Product, Sartorius, Stonehouse, Gloucestershire, UK) with different cut-offs (30, 10, 5 kDa). 20 mL of the samples were firstly loaded onto 30 kDa cut-off columns and centrifuged at 5000 rpm, room temperature, following manufacturer’s instructions. The permeate was loaded onto 10 kDa cut-off columns and the permeate successively loaded onto the 5 kDa cut-off columns. Retentate and permeate fractions were collected for further analysis and stored at -20°C.

### Determination of antioxidant activity

Antioxidant activity was measured using the ABTS (2,2'-azino-bis (3-ethylbenzothiazoline-6-sulphonic acid)) method [31]. 1 mL of the ABTS working solution was added to different aliquots of the samples (or the standard), and after incubation at 30°C in the dark for 30 minutes, Abs was measured at 734 nm. The results were expressed as grams of ascorbic acid (AA) equivalents per litre (gAA eq/L) by means of the dose-response calibration curve (between 0 and 2 μg of AA).

### Determination of anti-tyrosinase activity

Anti-tyrosinase activity was measured by an optimised tyrosinase inhibition assay described by Ferri et al. [14]. The results were expressed as grams of kojic acid (KA) equivalents per litre (gKA eq/L) by means of a dose-response calibration curve (between 0 and 10 μg of KA).

### Quantification of free amino acid by HPLC

Free amino acid quantification was performed by means of Waters AccQ Tag Amino Acid Analysis Method kit (Waters Corporation, Milford, MA, USA) following manufacturer’s instructions. Amino acid derivatisation [32] with AccQ Tag reagents was carried out as follows: 20 μL of sample was mixed to 60 μL of AccQ Fluor Borate Buffer and 20 μL of AccQ Fluor Reagent and incubated at 55°C for 10 minutes. Amino acid derivatives were chromatographically identified on HPLC coupled with a fluorescence detector (HPLC, Jasco, Grobumstad, Germany; equipped with a spectrofluorometer Jasco 821-FP) by using a AccQ Tag Column (3.9 mm x 150 mm, 4 μm, Waters AccQ Tag Amino Acid Analysis Column). Examples of amino acid separation chromatograms are reported in S2A–S2D Fig. The detection wavelength was set at 250 nm (λ excitation)– 395 nm (λ emission). A flow rate of 1 mL/min and a column temperature of 37°C were used. A standard mixture (Waters Amino Acid Hydrolysate Standard) of 17 free amino acids (serine, asparagine, histidine, glutamic acid, glycine, arginine, threonine, alanine, proline, cysteine, tyrosine, valine, methionine, lysine, isoleucine, leucine, phenylalanine; tryptophan, glutamine and asparagine not included) each one at concentration of 2.5 mM except for cystine (1.25 mM), was derivatised and, after HPLC separation, used for identification and quantification. Eluents: Waters AccQ Tag Eluent A (aqueous buffer) and eluent B (60:40 acetonitrile:water) and the following percentages of eluent A were used: 0 min 100%, 0.5 min 98%, 15 min 93%, 19 min 90%, 32 min 67%, 34 min 0%, 40 min 0%, 47 min 100%, 50 min 100%. Each run was of 50 minutes.

### Peptide identification

The two most promising fractions (see section 2.3) in terms of antioxidant and anti-tyrosinase activities were analysed using low resolution mass spectrometry with a reverse phase ultra-high performance liquid chromatography (UHPLC) coupled to electrospray ionization tandem mass spectrometry (ESI-MS/MS) (see an example of UHPLC separation chromatogram in S2E Fig) and high resolution tandem mass spectrometry with μHPLC coupled to Orbitrap LTQ XL mass spectrometer. Samples were centrifuged at 12,000 rpm for 10 min at 4°C and the clear supernatant was used for the analysis. Peptides were separated by reverse phase liquid chromatography and identified using both high and low resolution tandem mass spectrometry, as described in Prandi et al. [33]. The relative quantification of the peptides was obtained by direct comparison of the peak areas and of the -10lgP values, which are program scores indicating the quality of the identification. In order to reduce to zero the risk of false positives, positive hits for protein identification was set with -10lgP > 50.

### Statistical analyses

All the digestions were performed twice (biological replicates) and each repetition was analysed in two technical replicates. The results are expressed as the mean (n = 2) ± SD. All the statistical analyses were performed using R software version 1.3.5 (R Core Team, Vienna, Austria). Data were tested for normality using Shapiro-Wilk normality test and for homogeneity using the Levene's Test for Homogeneity of Variance with default parameters from the package *car* (https://CRAN.R-project.org/package=car). Since data resulted parametric, the analysis of variance test (ANOVA), followed by Tuckey’s multiple pairwise comparison post-hoc test (*p* < 0.03), were used to evaluate the differences among compared groups.

## Results and discussion

### Characterization of initial scotta and ultrafiltration samples

Table 2 reports total protein and dry weight levels of scotta (initial by-product), retentate 1 and retentate 2 samples (S, R1 and R2) obtained after two ultrafiltration steps from each scotta batch. In general, as expected, all R1 samples had a higher amount of proteins compared to S and R2. Batch 1 and 2 samples were obtained with the same ultrafiltration membrane cut-offs (Table 1) and, after comparison, batch 1 showed on average 2-fold higher amount of proteins than batch 2. This was probably due to differences of the initial provided scotta (S), which came from different harvest seasons and production sites (Table 1), influencing the original composition of milk. On average R1 samples of batch 1 and 2 had respectively 12 and 28-fold higher protein content than S and R2, while in batch 3 ad 4, R1 showed respectively10 and 2-fold higher protein amounts than S and R2; R1 from batch 5 had 27-fold higher protein content than S.

In general, S samples showed comparable levels of dry weight (43 to 54 g/L) with the exception of batch 2 (63 g/L) (Table 2). In accordance with the different membrane cut-offs used during the first ultrafiltration step, the dry weight content of R1 fractions of batch 1 and 2, was 1.6-fold higher than in batch 3 and 4. The same applies for R2 samples with batch 1, 2 and 3 fractions obtained with the same membrane cut-off (Table 1) (average of 63.9 g/L) and R2 from batch 4 being 2.2-fold higher (Table 2) resulting from a lower cut-off membrane in the second ultrafiltration step (4 kDa, Table 1) (138.5 g/L). The dry weight increase observed in R1 and R2 with respect to the initial scotta (Table 2) can be ascribed to sample concentration, while most of the aqueous phase passed through the membranes into the permeate. This fact was previously demonstrated for scotta in similar fractionation processes in which fat fraction was completely retained in R1 retentate [2].

### Optimisation of protease treatments: Digested protein yield and antioxidant activity

In preliminary digestion trials, nine different proteases were tested on S, R1, R2 samples of batch 1, by using three different enzyme/substrate ratios (E/S) at previously described conditions. Total proteins and antioxidant activity were measured in all digestates (S1 Table) and the molecular mass distribution of digestates was checked by SDS-PAGE (S1 Fig). Overall, the highest amount of hydrolysed proteins was determined in R1 digestate obtained with 5% Chymotrypsin (149.2 gBSA eq/L). S, R1 and R2 samples treated with 5–10% Chymotrypsin showed the most relevant antioxidant activity (up to 22-fold higher than in 37°C not digested (ND) control in R1 digested with 5% Chymotrypsin) while other enzymes produced samples having a maximum 5-fold increased antioxidant capacity in comparison to related ND controls (S1 Table).

Based on these preliminary data, the overall four best performing proteases were identified, and for each enzyme the best digestion conditions for S, R1 and R2 samples, yielding significantly higher protein amounts when compared with other protease treatments and same temperature ND controls (S1 Table), were selected. These were respectively 10% Bromelain, Pancreatin, Chymotrypsin and Papain for S and R2 samples, and 5% Pancreatin, Chymotrypsin and 10% Bromelain, Papain for R1 samples (S1 Table, data in bold).

Only for the selected hydrolysis conditions, 1, 2 and 4 h of incubation were tested, evidencing no significant difference in relation to increased incubation time and leading to select 1 h for the following process optimization steps.

**Fig 1:**
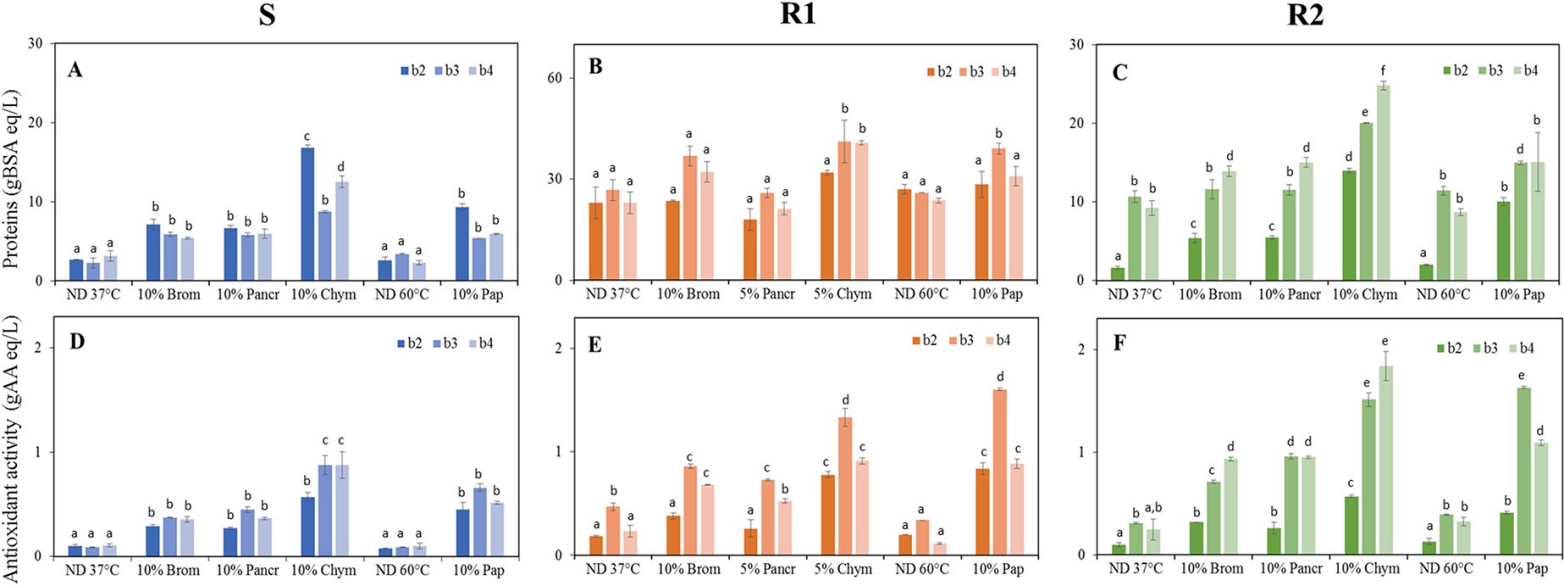
Quantification of total protein contents (A-C) and antioxidant activity levels (D-F) in hydrolysated samples (1h of incubation at optimal temperature) of batch 2 (b2), batch 3 (b3) and batch 4 (b4). S, scotta; R1; retantate 1; R2, retantate 2. Brom, bromelain; Pancr, pancreatin; Chym, chymotrypsin; Pap, papain; ND, not digested control. Different letters indicate statistically significant difference among samples of the same type (S, R1 or R2) determined by one way ANOVA followed by post-hoc Tuckey’s multiple pairwise comparison (*p* < 0.03). Data are the mean ± SD (n = 2).

The previous digestion conditions (enzyme type, E/S ratio and incubation time) were successively applied to S, R1 and R2 samples of batches 2 to 4 and total protein content (Fig 1A–1C) and antioxidant activity level (Fig 1D–1F) were measured. In general, batch 2–4 scotta digestates showed a slightly lower protein amount respect to batch 1, with the exception of batch 2 Chymotrypsin and Papain digestates (Fig 1A and S1 Table), this in accordance with the lower level of total proteins initially present in batch 2–4 feedstocks compared to batch 1 (Table 2).

No significant difference was generally shown on protein yield of S samples treated with Bromelain and Pancreatin with an average of 6.1 gBSA eq/L, 2.3-fold higher respect to 37°C ND, while Chymotrypsin digestates showed an average 2-fold higher protein content when compared to other enzymes (Fig 1A). R1 batch 2–4 samples showed on average a 2-fold higher amount of digested proteins respect to S and R2, with batch 3 and 4 having the highest level in all protease treatments independently from the membrane cut-off used during the first ultrafiltration step (Table 2). In general, Chymotrypsin and Papain were the best performing enzymes (Fig 1B). In accordance with initial amount of total proteins (Table 2), R2 digestates from batch 3 and 4 showed average protein levels 2-fold higher than batch 2. This could also be ascribed to the different membrane cut-off utilised for the second ultrafiltration step (Table 1) that led to the presence in R2 fractions, of proteins with a wider molecular weight range in batch 3 and 4 (500–30 kDa batch 3; 500–4 kDa batch 4) respect to batch 2 (100–30 kDa).

Antioxidant activity of batch 2–4 digestates was also detected (Fig 1D–1F). Among S samples, treatments with 10% Chymotrypsin showed the highest antioxidant capacity (on average 1.9-fold higher than other enzymes). In accordance with protein levels, in R1 and R2 samples the highest antioxidant capacity was detected respectively in batch 3 and 4 hydrolysates obtained with Chymotrypsin and Papain (Fig 1E and 1F). Antioxidant activities of scotta digestates were in the same order of magnitude of data obtained with similar protease digestion processes from rice protein by-product [14].

The amount of free amino acids was also measured in 20 mL digestates of batch 3 and 4 fractions after AccQ Tag kit derivatisation (S2 Table and S2 Fig). As expected in both batches, all hydrolysed samples showed higher amount of free amino acids than ND samples, with Pancreatin being the enzyme that generated the higher content of free amino acids (up to 21-fold higher in R1 batch 4 fraction compared to 37°C ND control).

Together with the involved industry and based on a balance between the amount of released peptides and the cost of the enzymes, Pancreatin and Papain were selected, even though they were not the best performing ones, for following scale up experiments. In fact, nonetheless Chymotrypsin was overall the most efficient enzyme, its cost, when considering the related process scaled up at an industrial size, seems not affordable and would lead to obtain a final peptide-based ingredient largely out the threshold of the actual benchmark of similar products.

**Fig 2:**
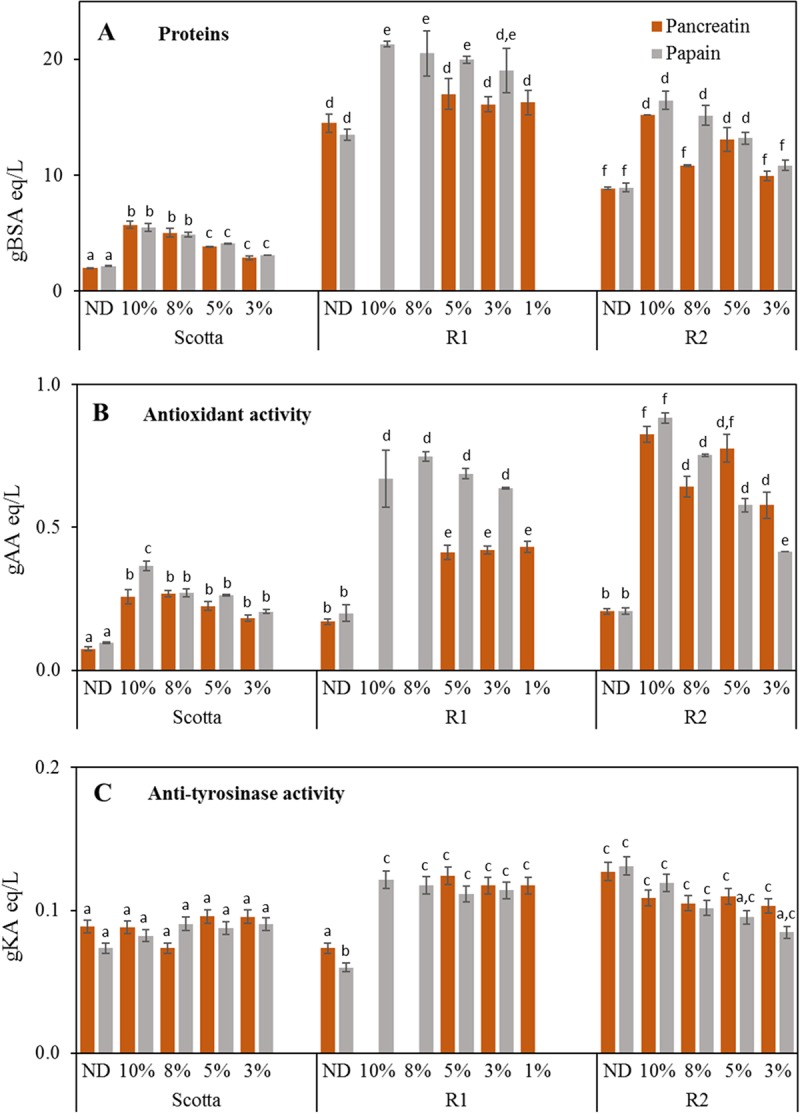
Quantification of total proteins (A), antioxidant activity (B) and anti-tyrosinase activity (C) in 200 mL Pancreatin and Papain digestions of batch 4 samples at different E/S ratios. S, scotta; R1; retantate 1; R2, retantate 2. ND, not digested controls at 37°C or 60°C. Different letters indicate statistically significant difference among the same assay (protein quantification, antioxidant activity, anti-tyrosinase activity) determined by one way ANOVA followed by post-hoc Tuckey’s multiple pairwise comparison (*p* < 0.03). AA, ascorbic acid; KA, kojic acid. Data are the mean ± SD (n = 2).

In this view, following experiments were aimed at establishing the minimum enzyme amount needed to achieve the highest release of bioactive peptides. Digestion protocols were 10-fold scaled up (200 mL total reaction volume) by using batch 4 samples (Fig 2) and hydrolyses were performed with decreasing E/S ratios starting from the maximum enzyme amount previously selected (Fig 1) (from 10% to 1% depending on treated sample and previously chosen conditions).

In all samples, a general decrease of protein yield (Fig 2A) as well as of antioxidant activity (Fig 2B), was measured in agreement with the E/S ratio, with the exception of R1 fraction treated with Pancreatin which did not show any variation (on average 16.46 gBSA eq/L and 0.42 gAA eq/L). Anti-tyrosinase activity was present in all samples with no significant difference between the two enzyme treatments (Fig 2C); in comparison with ND controls, only R1 hydrolysates showed a significant activity increase, while a gradual decrease was detected in R2 samples depending on E/S ratio.

Free amino acids content measured in batch 4 200 mL Pancreatin hydrolysates (S2 Table) showed from 10 to 41-fold higher levels than 37°C ND. Overall, the highest free amino acid increase was obtained in R2 sample treated with 5% Papain compared to 60°C ND (47-fold). In batch 4 at 200 mL scale, the more abundant amino acids were histidine, arginine, threonine, cysteine, tyrosine, lysine and leucine, of which histidine, threonine and lysine are essential amino acids with histidine being also a functional amino acid [34], tyrosine is a precursor of many important biological compounds essential for the functioning of human organism [35], leucine has an important role in intracellular signalling [36] and cysteine is a nutritionally semi-essential amino acid [37]. These data seems to support the potential exploitation of scotta digestates in the food sector.

Based on previous results and taking again into consideration the foreseen industrial process costs, the following conditions, aimed at obtaining the highest amount of bioactive peptides, were selected as final digestion conditions for S, R1 and R2 samples: 3% Pancreatin or 5% Papain, 1h of incubation at 37°C or 60°C respectively.

### Single step ultrafiltration process and scale up

In order to improve the protein yield, a single ultrafiltration step was performed on initial batch 5 scotta feedstock using a lower pore size cut-off membrane (10 kDa, Table 2). As expected, the resulting R1 fraction showed a 2.6–1.8 fold increased protein and dry weight contents when compared to R1 fractions of batches 2–4 (Table 2).

**Fig 3:**
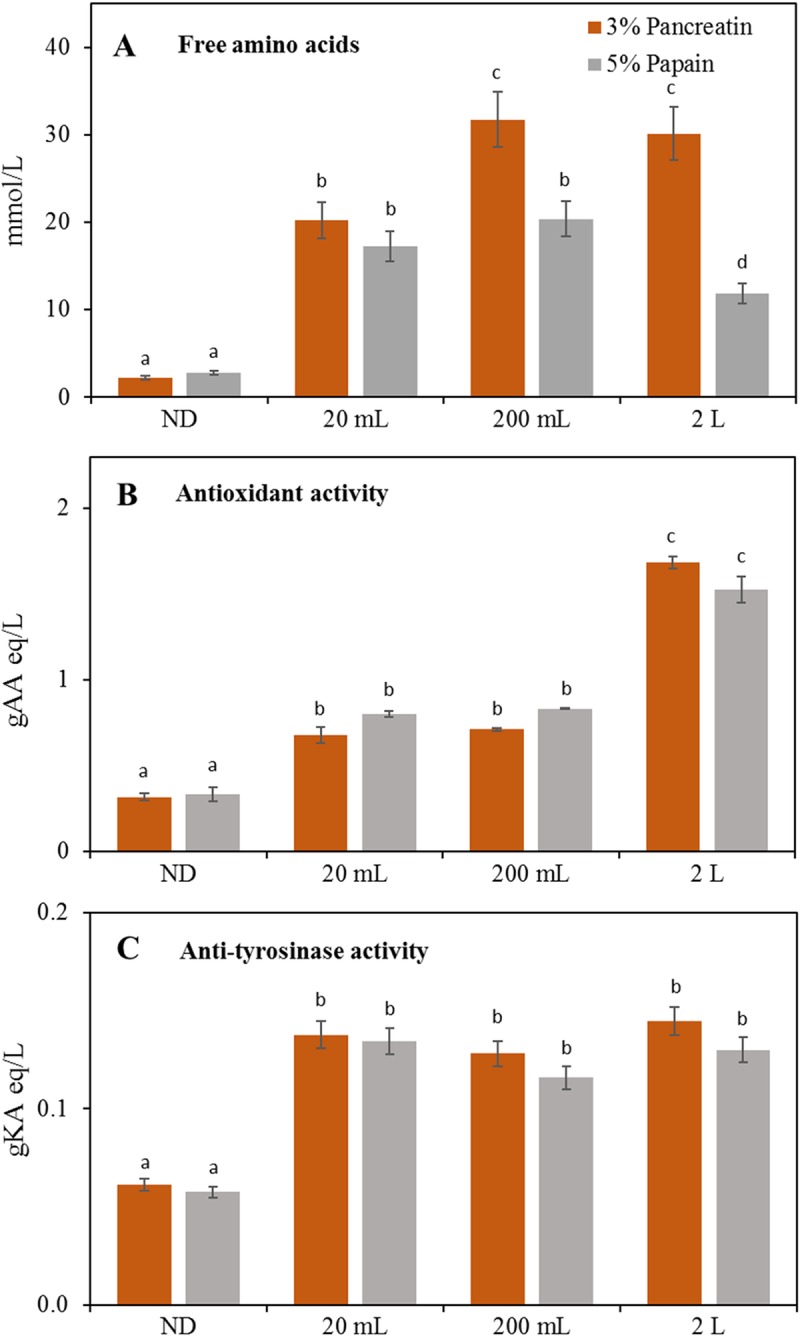
Quantification of free amino acids (A), antioxidant activity (B) and anti-tyrosinase activity (C) in different volume digestions of batch 5 R1 fraction. ND, not digested controls at 37°C or 60°C. Different letters indicate statistically significant difference among the same assay (free amino acid quantification, antioxidant activity, anti-tyrosinase activity) determined by one way ANOVA followed by post-hoc Tuckey’s multiple pairwise comparison (*p* < 0.03). AA, ascorbic acid; KA, kojic acid. Data are the mean ± SD (n = 2).

The previously selected digestion conditions were successively applied to R1 fraction of batch 5 by comparing three different reaction volumes: 20 mL, 200 mL and 2 L (Fig 3).

The highest amount of digested proteins was detected in Pancreatin 2 L samples (60 gBSA eq/L) while no significant difference was observed between 20 mL and 200 mL digestions in both enzymatic treatments (average of 50 gBSA eq/L). In addition, an increased amount of free amino acids was determined in particular in 200 mL and 2 L Pancreatin digestates with an average 1.5–2.5-fold higher free amino acid levels respect to Papain samples (Fig 3A, S2 Table). These data led to conclude that, nonetheless the application of the same digestion conditions, the efficiency of the hydrolysis process increases with the scale due most probably to better stirring and, specifically for 2 L digestions performed in bioreactor, to a better pH control of the reaction. Taking into consideration that the Lowry method applied for the quantification of total digested proteins, can only detect intact proteins or peptides and does not measure free amino acids [38], the amino acid levels seem to be in accordance with total protein data and show opposite trends in the two enzyme treatments (i.e. high free amino acids and low total proteins in Pancreatin and viceversa in Papain). This data could be explained considering the mechanism of action of the two proteases. In fact, Pancreatin is a mixture of enzymes among which proteases which can break down proteins into monomers starting from the terminal amino acids, while Papain is a cysteine endopeptidase that breaks peptide bonds of nonterminal amino acids, therefore mostly producing peptides and not free amino acids [39]. The amino acids mostly released in batch 5 at all digestion volumes are cysteine, proline, tyrosine, lysine and leucine; particularly worth of notice was the content of cysteine which, in particular after Pancreatin hydrolysis, was 8 to 12-fold higher than the average of other amino acids (S2 Table).

In agreement with previous results, antioxidant activity in 20 mL and 200 mL digestions showed in both treatments an average 2.8-fold higher activity than ND controls. The highest activity was measured in 2 L digestions (average of 6.0-fold increase respect to ND) (Fig 3B). Total antioxidant capacity were similar in 2 L Pancreatin and 2 L Papain samples (Fig 3B), while free amino acids were 2.5-times lower in Papain digestate (Fig 3A). Moreover, cysteine was the most abundant free amino acid released by both the enzymes, between 1.4 and 2.1-times more in Pancreatin digestates with respect to Papain (S2 Table, batch 5 data), and cysteine exerts a well-known antioxidant activity due to the ability of thiols to undergo redox reactions [23, 37]. Considered together, these data again indirectly demonstrate the higher digestion efficiency in 2 L samples with the presence of a large amount of peptides especially in Papain samples. In fact, in accordance with previous studies, small molecular weight food-derived peptides exert a higher antioxidant activity respect to free amino acids [40].

Anti-tyrosinase activity increased on average of 2-fold in all digested samples compared to ND, with no specific difference based on type of enzyme treatment or digestion volume (Fig 3C). This is the first work in which anti-tyrosinase activity was detected in animal dairy-based hydrolysed peptides. When compared with plant hydrolysed peptides [14], the present samples showed 3.2-times higher anti-tyrosinase activity than rice digestates obtained with Alcalase and Neutrase and 2.4-times lower activity than Protamex-hydrolysed samples. This is probably due to the different amino acid composition of animal and plant proteins and the consequent release of peptides with different sizes and bioactivities.

### Sample fractionation

**Fig 4:**
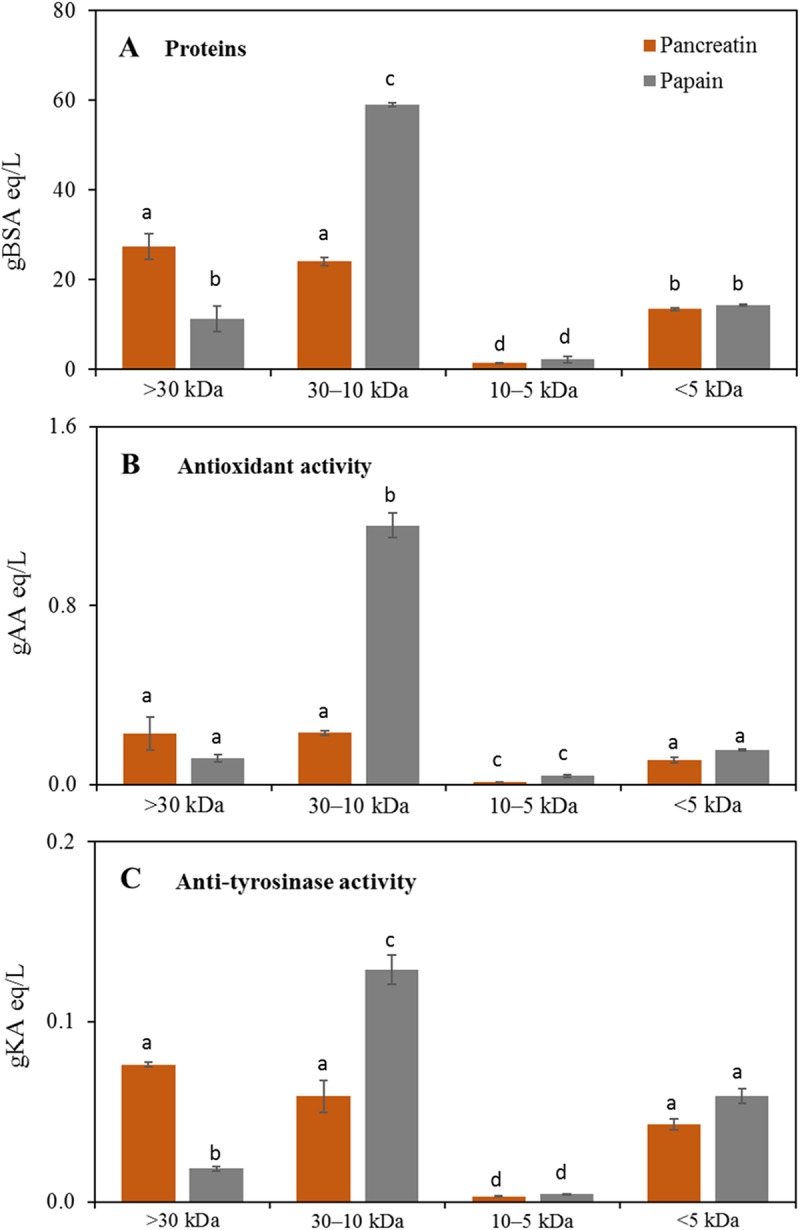
Quantification of total proteins (A), antioxidant activity (B) and anti-tyrosinase activity (C) in molecular weight protein fractions obtained after sequential centrifugation with different cut-off membranes of R1 batch 5, 2 L Pancreatin and Papain digestates. Different letters indicate statistically significant difference among the same assay (protein quantification, antioxidant activity, anti-tyrosinase activity) determined by one way ANOVA followed by post-hoc Tuckey’s multiple pairwise comparison (*p* < 0.03). AA, ascorbic acid; KA, kojic acid. Data are the mean ± SD (n = 2).

The 2 L digestates were also sequentially fractioned by means of centrifugation columns with different cut-offs (30, 10, 5 kDa) and retentate and permeate fractions obtained from the two selected enzymes were analysed (Fig 4).

The highest amount of yielded proteins were recovered for Pancreatin treatment in > 30 kDa and 30–10 kDa fractions, and for Papain in the 30–10 kDa retentate, which was overall the fraction having the major content of proteins (59.0 gBSA eq/L), about 2.4-fold higher than the correspondent Pancreatin fraction (Fig 4A). The fractions 10–5 kDa and < 5 kDa showed a lower amount of proteins without any significant difference between the two enzymatic treatments. Similarly to the protein content, also antioxidant (Fig 4B) and anti-tyrosinase (Fig 4C) activities showed their maximum levels in Papain 30–10 kDa fraction (respectively 1.16 gAA eq/L and 0.14 gKA eq /L). R1 hydrolysed fractions bioactivities levels were of the same order of magnitude but with a different distribution among the fractions, of previously reported rice peptides [14]. In particular, the most active fraction (Papain 30–10 kDa) had the same antioxidant capacity and a 2-fold higher anti-tyrosinase activity with respect to the average of Neutrase and Alcalase analogous rice fractions. On the contrary, Pancreatin digested fractions showed 4- and 3-fold lower activities, respectively, when compared with the best rice fractions with a similar molecular weight range (Protamex, > 8 kDa and 8–5 kDa) [14].

### Peptide characterization

The two most promising fractions in terms of both antioxidant and anti-tyrosinase activities (Papain 30–10 kDa and, to a lesser extent, Papain < 5 kDa fraction, Fig 4B and 4C) were analysed by both high and low resolution tandem mass spectrometry in order to obtain the peptide sequences.

Chromatograms of the two samples showed a similar trend and, by comparison, the peaks corresponding to the most abundant peptide species were selected by obtaining the relative quantification of the peptides (by direct comparison of the peaks areas) and identified by using Peaks Studio database (see an example of chromatogram in S2E Fig). As shown in Table 3, most of the peptides having the highest score (-10lgP) value derived from β-casein and α-lactalbumin. Several octameric peptides, derived from the same type of milk proteins, were previously found to be able to bind and to inhibit tyrosinase, thanks to the presence of arginine and/or phenylalanine in combination with valine, alanine and/or leucine, in their amino acid sequence [28]. The same five amino acids were largely present in all the peptides identified in the present study (Table 3).

**Table 3:** Peptides identified with liquid chromatography coupled with high resolution mass spectrometry.

**Papain, fraction 30–10 kDa**								
Peptide	-10lgP	Mass (Da)	Length	m/z	RT	Intensity	Accession	PTM
SEEQQQTEDELQDKIHPFA	82.14	2271.0291	19	1136.524	25.3	9.41E+07	β-casein	
DKFLDDDLTDDIM(+15.99)	81.56	1570.6708	13	786.3439	28.5	1.52E+08	α-lactalbumin	Oxidation (M)
Q(-17.03)TEDELQDKIHPFAQ	77.76	1780.8268	15	891.421	26.4	3.34E+07	β-casein	Pyro-glu (from Q)
DKFLDDDLTDDIMC(+119.00)	76.84	1776.6892	14	889.353	30.8	8.22E+07	α-lactalbumin	Cysteinylation
K(+114.04)FLDDDLTDDIM	76.45	1553.6919	12	777.8535	31.5	2.58E+07	α-lactalbumin	Ubiquitin
TEDELQDKIHPFA	75.67	1541.7361	13	771.8761	24	1.51E+08	β-casein	
KTVDMES(+79.97)TEVFTK	74.74	1593.6997	13	797.8574	21.6	1.48E+07	α_S2_-casein	Phosphorylation (STY)
DKFLDDDLTDDIMC(+305.07)V	74.32	2061.8218	15	1031.928	33.9	2.49E+08	α-lactalbumin	Glutathione disulfide
DKFLDDDLTDDIMC(+133.02)V	72.64	1889.7733	15	945.8951	31.5	1.40E+07	α-lactalbumin	S-homocysteinylation
EEQQQTEDELQDKIHPFA	70.57	2183.9971	18	1093.007	24.9	6.98E+06	β-casein	
DKFLDDDLTDDIMC(+267.03)VK	70.4	2151.8799	16	1076.944	35.1	1.26E+08	α-lactalbumin	Nitroso Sulfamethoxazole Sulfinamide thiol adduct
DKFLDDDLTDDIMC(+119.00)VK	70.1	2003.8525	16	1002.935	28.9	3.03E+07	α-lactalbumin	Cysteinylation
KFQS(+79.97)EEQQQTEDELQDKIHPF	70.05	2683.1802	21	895.4025	26.7	6.06E+07	β-casein	Phosphorylation (STY)
**Papain, fraction <5 kDa**								
Peptide	-10lgP	Mass (Da)	Length	m/z	RT	Intensity	Accession	PTM
DKFLDDDLTDDIM(+15.99)	85.45	1570.6708	13	786.3438	28.4	6.11E+07	α-lactalbumin	Oxidation (M)
Q(-17.03)QQTEDELQDKIHPFA	72.32	1908.8854	16	955.4519	27.1	1.32E+07	β-casein	Pyro-glu (from Q)
DKFLDDDLTDDIM	71.94	1554.6759	13	778.3455	31.9	2.61E+07	α-lactalbumin	
YPFPGPIHN(+.98)SLPQ	68.73	1466.7194	13	734.3683	28.5	5.21E+07	β-casein	Deamidation (NQ)
Q(-17.03)TEDELQDKIHPFAQ	66.45	1780.8268	15	891.4222	26.4	1.55E+07	β-casein	Pyro-glu (from Q)
DKFLDDDLTDDIMC(+305.07)V	65.91	2061.8218	15	1031.928	33.9	4.42E+07	α-lactalbumin	Glutathione disulfide
DELQDKIHPFA	63.65	1311.6459	11	656.8309	24.1	6.43E+08	β-casein	
Q(-17.03)TEDELQDKIHPF	63.5	1581.7311	13	791.8743	27.4	2.86E+07	β-casein	Pyro-glu (from Q)
DK(+14.02)FLDDDLTDDIM	62.12	1568.6915	13	785.3544	31.9	6.95E+06	α-lactalbumin	Methylation(KR)
KFLDDDLTDDIM(+15.99)	61.63	1455.6439	12	728.8304	27.9	2.25E+07	α-lactalbumin	Oxidation (M)
Q(-17.03)TEDELQDKIHPFA	60.21	1652.7682	14	827.3926	27.2	1.40E+08	β-casein	Pyro-glu (from Q)
DKFLDDDLTDDIMC(-2.95)V	59.88	1753.808	15	877.9135	29.4	4.20E+06	α-lactalbumin	Carbamidomethylated Cys that undergoes beta-elimination and Michael addition of methylamine
KPEDETHLE	59.86	1096.5037	9	549.2592	12.9	1.61E+08	GLYCAM1	

-10lgP is a score indicating the quality of the identification, m/z is the mass to charge ratio of the fragmented ion, PTM, Post Translational Modification, indicates the type of modification and amino acid (in brackets) on which the modification occurs.

Interestingly, the identified peptides could be ascribed to very specific protein regions: in the case of α-lactalbumin, peptides came from the region comprised between amino acid residues 97 and 112. Peptides from α-lactalbumin contain sulphur amino acids, one methionine and one cysteine, which also present disulphide bond linking to the cysteine residue 92 (cystine peptides). The thiol, in cysteine, and the thioether, in methionine, are relatively easily oxidized [41], thus exerting a protective effect against oxidation of other substrates. The presence of these two amino acid residues could be responsible for the positive results observed for both antioxidant and anti-tyrosinase activities [13, 42]. To this extent, it has been recently reported the possible applications of buffalo whey hydrolysed with Alcalase as a natural substitute for additives conventionally used in the control of enzymatic browning in foods [43]. Moreover, it has been reported that glutathione and some glutathione analogues promoted tyrosinase inactivation; in particular, the addition of the -CH_2_SH moiety significantly improved the inhibition activity over the parent molecule [44]. Thus, the high presence of peptides containing sulphur amino acids (as shown in Table 3) could explain the anti-tyrosinase activity of these two scotta fractions. These regions of α-lactalbumin also show high amount of aspartic acid residues. In the case of β-casein, the peptides were obtained from a quite restricted protein region, between the 47^th^ and the 69^th^ residue, which is rich in glutamic acid and glutamine residues. The side chains of acidic amino acids and their analogues are effective against peroxynitrite [45], thus possibly exerting an antioxidant activity. In case of many plant extracts, fractions and compounds (e.g. grape polyphenols, rice protein digestates) that exert an antioxidant activity, were reported to have also an anti-tyrosinase activity [14, 25, 42]. It is not surprising therefore, to find that both antioxidant and anti-tyrosinase activities were present in the same scotta sub-fractions, leading to hypothesise that the same peptides could be responsible for both biological effects.

## Conclusions

In the present work, scotta feedstock, from ricotta cheese industrial production, was collected and, after ultra-filtration, the enriched protein fractions were for the first time used in order to optimise enzyme-based valorisation protocols. At first, nine different commercial food-grade proteolytic enzymes were tested on scotta, Retentate 1 and Retentate 2 ultra-filtration fractions. Based on digested protein yields, fraction bioactivities and foreseen scale up processing costs, 3% Pancreatin or 5% Papain (37°C or 60°C respectively, 1 h incubation) were identified as the best hydrolysis conditions and 10 and 100-fold volume scale-up of enzymatic digestion processes were set up. The digestion efficiency increased with the reaction volume (up to 60 gBSA eq/L) as well as antioxidant activity (up to 1.7 gAA eq/L). Hydrolysed proteins were also sub-fractionated by means of physical membrane-based separations at decreasing cut-offs and sub-fractions tested for their biological activities. In particular, Papain treatment produced relatively high amounts of low molecular weight peptides showing, *in vitro*, antioxidant (up to 1.16 gAA eq/L) and, for the first time on dairy-derived peptides, anti-tyrosinase (up to 0.14 gKA eq/L) biological activities. The peptide sequence of the most bioactive fractions was achieved. As no chemicals or solvents were used all along the process, the present results suggest that a future direct exploitation of isolated peptide fractions from the industrial scotta by-product in the nutraceutical, functional food, feed and cosmetic industrial fields, is feasible and could be at short term implemented.

## Supporting information

S1 Table Content of total proteins (gBSA eq/L) and antioxidant activity expressed as g of ascorbic acid (AA) equivalent per L (gAA eq/L), of digested (2h of incubation at optimal temperature) and not digested (ND) batch 1 samples. S, scotta; R1; retentate 1; R2, retentate 2. Different letters indicate statistically significant difference among samples of the same type (S, R1 or R2, for the same analysis) determined by one way ANOVA followed by post-hoc Tuckey’s multiple pairwise comparison (*p* < 0.03). Data are the mean ± SD (n = 2). In bold treatments selected for further experiments.
(DOCX)

S2 Table Quantification of free amino acids in digested and not digested (ND) control scotta (S), rententate1 (R1) and retentate 2 (R2) samples of batches 3, 4 and 5 at 20 mL, 200 mL and 2 L treatment volumes. Amino acids content (μmol/L) was measured by HPLC-fluorimeter after AccQ Tag kit derivatisation. Data are the mean of two independent analyses and a 15% SD applies to all values.
(DOCX)

S1 Fig **Example of molecular mass distribution (mono-dimensional SDS-PAGE) of initial protein fractions (scotta (A) and retentate 1 (B)) and related peptides obtained after different protease treatments of batch 4 (20 mL incubation volumes).** Pancr, pancreatin; Brom, bromelain; Chym, chymotrypsin; Pap, papain; ND, not digested control.
(TIF)

S2 Fig Examples of HPLC-fluorimeter and reverse phase UHPLC/ESI-MS chromatographic separation of digested protein fractions. **(A-D)** HPLC-fluorimeter chromatograms reporting the identification of free amino acids after Pancreatin digestion of initial fractions (20 mL incubation volume). Amino acids standards (A), Scotta (10% Pancreatin, 1h 37°C) (B), Retentate 1 (5% Pancreatin 1 h 37°C) (C), Retentate 2 (10% Pancreatin 1h 37°C) (D). Amino acids: ser (1), asp (2), his (3), glu (4), gly (5), arg (6), thr (7), ala (8), pro (9), cys (10), tyr (11), val (12), met (13), lys (14), ile (15), leu (16), phe (17). **(E) Reverse phase UHPLC/ESI-MS chromatogram (Full Scan)** of < 5 kDa fraction obtained from retentate 1 sample hydrolysed with 5% Papain 1h at 60°C.
(TIF)

## Abbreviations

AA: ascorbic acid
ABTS: 2,2'-azino-bis (3-ethylbenzothiazoline-6-sulphonic acid)
DW: dry weight
E/S: enzyme/substrate ratio
HPLC: high-performance liquid chromatography
KA: kojic acid
ND: not digested
R1: retentate 1
R2: retentate 2
S: scotta
SD: standard deviation
UHPLC: ultra high performance liquid chromatography
