# Supplementary material for: Enzymatic production of bioactive peptides from scotta, an exhausted by-product of ricotta cheese processing
Source: PLoS One. 2019 Dec 30;14(12):e0226834. doi: 10.1371/journal.pone.0226834 (PMC6936807; doi:10.1371/journal.pone.0226834)
Supplement: S1 Table — S, scotta; R1; retentate 1; R2, retentate 2. Different letters indicate statistically significant difference among samples of the same type (S, R1 or R2, for the same analysis) determined by one way ANOVA followed by post-hoc Tuckey’s multiple pairwise comparison (p < 0.03). Data are the mean ± SD (n = 2). In bold treatments selected for further experiments. (DOCX) [file pone.0226834.s001.docx]

**S1 Table.** **Content of total proteins (gBSA eq/L) and antioxidant activity expressed as g of ascorbic acid (AA) equivalent per L (gAA eq/L), of digested (2h of incubation at optimal temperature) and not digested (ND) batch 1 samples**. S, scotta; R1; retentate 1; R2, retentate 2. Different letters indicate statistically significant difference among samples of the same type (S, R1 or R2, for the same analysis) determined by one way ANOVA followed by post-hoc Tuckey’s multiple pairwise comparison (*p* < 0.03). Data are the mean ± SD (n =2). In bold treatments selected for further experiments.

| **Enzyme** | **E/S ratio (%, w/w)** | **Total proteins (gBSA eq/L)** | | | **Antioxidant activity (gAA eq/L)** | | |
| --- | --- | --- | --- | --- | --- | --- | --- |
|  |  | **S** | **R1** | **R2** | **S** | **R1** | **R2** |
| ND 37°C | - | 3.8 ± 0.8 ^a^ | 65.4 ± 9.7 ^a,b^ | 1.0 ± 0.4 ^a^ | 0.08 ± 0.01 ^a^ | 0.15 ± 0.01 ^a^ | 0.11 ± 0.05 ^a^ |
|  | 1 | 5.1 ± 0.1 ^b^ | 33.2 ± 15.2 ^c^ | 2.3 ± 0.1 ^b^ | 0.11 ± 0.02 ^a^ | 0.45 ± 0.15 ^b^ | 0.11 ± 0.03 ^a^ |
| Bromelain | 5 | 7.0 ± 0.4 ^c^ | 66.5 ± 0.0 ^a^ | 4.7 ± 0.6 ^d^ | 0.15 ± 0.04 ^a^ | 0.54 ± 0.12 ^b^ | 0.17 ± 0.02 ^a^ |
|  | 10 | **8.7 ± 0.3** ^d^ | **78.9 ± 1.4** ^b^ | **6.8 ± 0.2** ^e^ | 0.21 ± 0.03 ^b^ | 0.65 ± 0.15 ^b^ | 0.22 ± 0.05 ^a^ |
|  | 1 | 3.4 ± 0.3 ^a^ | 78.3 ± 2.0 ^a,b^ | 1.2 ± 0.2 ^a^ | 0.13 ± 0.01 ^a^ | 0.52 ± 0.11 ^b^ | 0.11 ± 0.03 ^a^ |
| Pancreatin | 5 | 4.9 ± 0.4 ^b^ | **85.0 ± 4.6** ^b^ | 3.0 ± 0.1 ^c^ | 0.15 ± 0.03 ^a^ | 0.63 ± 0.06 ^b^ | 0.16 ± 0.04 ^a^ |
|  | 10 | **6.5 ± 0.1** ^c^ | 60.8 ± 6.4 ^a^ | **4.5 ± 0.5** ^c^ | 0.16 ± 0.02 ^a^ | 0.55 ± 0.08 ^b^ | 0.21 ± 0.05 ^a^ |
|  | 1 | 5.4 ± 0.3 ^b^ | 139.5 ± 8.9 ^d^ | 4.0 ± 0.1 ^c^ | 0.84 ± 0.08 ^b^ | 2.72 ± 0.40 ^c^ | 0.47 ± 0.07 ^b^ |
| Chymotrypsin | 5 | 8.6 ± 0.3 ^d^ | **149.2 ± 3.9** ^d^ | 8.6 ± 0.1 ^e^ | 1.27 ± 0.13 ^b^ | 3.32 ± 0.06 ^c^ | 1.25 ± 0.07 ^c^ |
|  | 10 | **12.5 ± 0.1** ^e^ | 123.4 ± 7.8 ^d^ | **13.4 ± 0.2** ^f^ | 1.90 ± 0.03 ^b^ | 3.03 ± 0.16 ^c^ | 1.99 ± 0.07 ^d^ |
|  | 1 | 4.7 ± 0.2 ^b^ | 32.6 ± 0.1 ^c^ | 2.0 ± 0.1 ^b^ | 0.11 ± 0.02 ^a^ | 0.48 ± 0.02 ^b^ | 0.10 ± 0.03 ^a^ |
| Trypsin | 5 | 5.4 ± 0.3 ^b^ | 77.3 ± 2.4 ^b^ | 2.8 ± 0.1 ^b^ | 0.14 ± 0.02 ^a^ | 0.57 ± 0.05 ^b^ | 0.12 ± 0.03 ^a^ |
|  | 10 | 5.8 ± 0.1 ^b^ | 63.8 ± 3.9 ^a^ | 3.5 ± 0.1 ^c^ | 0.15 ± 0.03 ^a^ | 0.63 ± 0.09 ^b^ | 0.18 ± 0.03 ^a^ |
| ND 50°C | - | 5.4 ± 0.2 ^b^ | 66.6 ± 9.0 ^a,b^ | 2.1 ± 0.1 ^b^ | 0.09 ± 0.02 ^a^ | 0.13 ± 0.01 ^a^ | 0.10 ± 0.01 ^a^ |
|  | 1 | 5.3 ± 0.2 ^b^ | 67.6 ± 2.5 ^a^ | 3.1 ± 0.2 ^c^ | 0.14 ± 0.06 ^a^ | 0.29 ± 0.10 ^d^ | 0.13 ± 0.04 ^a^ |
| Neutrase 0.8L | 5 | 5.8 ± 0.1 ^b^ | 69.9 ± 7.1 ^a^ | 3.3 ± 0.1 ^c^ | 0.14 ± 0.03 ^a^ | 0.38 ± 0.09 ^d^ | 0.14 ± 0.03 ^a^ |
|  | 10 | 6.1 ± 0.1 ^b^ | 62.2 ± 3.3 ^a^ | 3.6 ± 0.3 ^c^ | 0.16 ± 0.03 ^b^ | 0.43 ± 0.12 ^b^ | 0.16 ± 0.04 ^a^ |
|  | 1 | 4.6 ± 0.1 ^b^ | 42.7 ± 4.0 ^e^ | 2.5 ± 0.1 ^b^ | 0.12 ± 0.02 ^a^ | 0.18 ± 0.12 ^a^ | 0.11 ± 0.02 ^a^ |
| Flavourzyme 500L | 5 | 4.1 ± 0.1 ^a^ | 34.7 ± 1.8 ^c^ | 2.8 ± 0.6 ^b^ | 0.15 ± 0.06 ^a^ | 0.24 ± 0.11 ^a,d^ | 0.12 ± 0.03 ^a^ |
|  | 10 | 3.7 ± 0.8 ^a^ | 41.1 ± 9.2 ^e^ | 2.9 ± 0.1 ^b^ | 0.12 ± 0.01 ^a^ | 0.31 ± 0.06 ^d^ | 0.14 ± 0.04 ^a^ |
| ND 60°C | - | 4.0 ± 0.1 ^a^ | 64.8 ± 6.6 ^a^ | 1.5 ± 0.1 ^a^ | 0.06 ± 0.01 ^a^ | 0.17 ± 0.02 ^a^ | 0.07 ± 0.01 ^e^ |
|  | 1 | 5.6 ± 1.4 ^b,c^ | 69.6 ± 3.1 ^a^ | 1.8 ± 0.1 ^a^ | 0.10 ± 0.04 ^a^ | 0.28 ± 0.12 ^d^ | 0.10 ± 0.06 ^a^ |
| Alcalase 2.4L | 5 | 4.8 ± 0.4 ^b^ | 75.1 ± 0.3 ^b^ | 2.3 ± 0.2 ^b^ | 0.11 ± 0.05 ^a^ | 0.33 ± 0.10 ^d^ | 0.10 ± 0.05 ^a^ |
|  | 10 | 3.9 ± 0.8 ^b^ | 73.5 ± 7.1 ^a,b^ | 2.5 ± 0.1 ^b^ | 0.11 ± 0.02 ^a^ | 0.39 ± 0.05 ^d^ | 0.09 ± 0.02 ^a^ |
|  | 1 | 4.3 ± 0.1 ^b^ | 43.3 ± 13.3 ^c,e^ | 1.7 ± 0.1 ^a^ | 0.12 ± 0.05 ^a^ | 0.24 ± 0.04 ^d^ | 0.09 ± 0.03 ^a^ |
| Protamex | 5 | 4.3 ± 0.8 ^b^ | 66.9 ± 0.6 ^a^ | 2.1 ± 0.4 ^a,b^ | 0.09 ± 0.01 ^a^ | 0.38 ± 0.04 ^d^ | 0.10 ± 0.03 ^a^ |
|  | 10 | 4.5 ± 1.2 ^b^ | 64.2 ± 0.7 ^a^ | 2.6 ± 0.1 ^b^ | 0.10 ± 0.04 ^a^ | 0.43 ± 0.06 ^b^ | 0.09 ± 0.02 ^a^ |
|  | 1 | 5.2 ± 0.2 ^b^ | 82.2 ± 6.4 ^b^ | 2.6 ± 0.4 ^b^ | 0.10 ± 0.02 ^a^ | 0.29 ± 0.10 ^a,d^ | 0.09 ± 0.02 ^a^ |
| Papain | 5 | 7.3 ± 0.2 ^c^ | 89.0 ± 6.8 ^b^ | 5.3 ± 0.1 ^c^ | 0.11 ± 0.01 ^a^ | 0.35 ± 0.07 ^b^ | 0.13 ± 0.04 ^a^ |
|  | 10 | **7.6 ± 1.9** ^c,d^ | **98.7 ± 3.5** ^b^ | **9.4 ± 0.3** ^e^ | 0.15 ± 0.02 ^b^ | 0.46 ± 0.10 ^b,d^ | 0.16 ± 0.03 ^a^ |
