# Supplementary material for: Enzymatic production of bioactive peptides from scotta, an exhausted by-product of ricotta cheese processing
Source: PLoS One. 2019 Dec 30;14(12):e0226834. doi: 10.1371/journal.pone.0226834 (PMC6936807; doi:10.1371/journal.pone.0226834)
Supplement: S2 Table — Amino acids content (μmol/L) was measured by HPLC-fluorimeter after AccQ Tag kit derivatisation. Data are the mean of two independent analyses and a 15% SD applies to all values. (DOCX) [file pone.0226834.s002.docx]

**S2 Table**. **Quantification of free amino acids in digested and not digested (ND) control scotta (S), rententate1 (R1) and retentate 2 (R2) samples of batches 3, 4 and 5 at 20 mL, 200 mL and 2 L treatment volumes.** Amino acids content (µmol/L) was measured by HPLC-fluorimeter after AccQ Tag kit derivatisation. Data are the mean of two independent analyses and a 15% SD applies to all values.

|  |  | **Sample** | **SER** | **ASP** | **HIS** | **GLU** | **GLY** | **ARG** | **THR** | **ALA** | **PRO** | **CYS** | **TYR** | **VAL** | **MET** | **LYS** | **ILE** | **LEU** | **PHE** | **Total Amount** |
| --- | --- | --- | --- | --- | --- | --- | --- | --- | --- | --- | --- | --- | --- | --- | --- | --- | --- | --- | --- | --- |
| **Batch 3** | **20 mL** |  | **Scotta** | | | | | | | | | | | | | | | | |  |
|  |  | 37°C control | 0.0 | 1.4 | 2.7 | 11.9 | 93.2 | 9.3 | 47.3 | 3.3 | 0.0 | 7.4 | 1.3 | 1.5 | 0.5 | 2.9 | 0.8 | 0.6 | 0.9 | 184.9 |
|  |  | 60°C control | 0.0 | 0.7 | 2.2 | 12.8 | 61.9 | 11.9 | 56.4 | 3.2 | 0.0 | 29.0 | 0.8 | 1.1 | 0.0 | 0.0 | 0.4 | 0.2 | 0.2 | 181.0 |
|  |  | 10% bromelain | 9.5 | 3.2 | 2.3 | 5.6 | 69.4 | 8.2 | 60.5 | 6.7 | 0.7 | 74.7 | 2.3 | 2.8 | 2.7 | 11.4 | 0.8 | 3.2 | 2.4 | 266.2 |
|  |  | 10% pancreatin | 34.0 | 16.7 | 14.8 | 43.1 | 81.4 | 56.5 | 56.7 | 23.0 | 2.4 | 155.7 | 37.3 | 18.5 | 4.4 | 121.9 | 19.1 | 37.7 | 21.4 | 744.7 |
|  |  | 10% chymotrypsin | 5.5 | 4.2 | 4.4 | 21.6 | 57.6 | 31.5 | 50.3 | 5.0 | 2.0 | 131.3 | 9.4 | 4.7 | 4.1 | 41.2 | 13.3 | 10.0 | 2.7 | 398.8 |
|  |  | 10% papain | 3.8 | 3.4 | 12.7 | 16.1 | 92.1 | 0.0 | 40.0 | 6.1 | 0.0 | 114.3 | 0.7 | 2.5 | 0.8 | 6.8 | 1.2 | 3.3 | 1.4 | 305.3 |
|  |  |  | **Retentate 1** | | | | | | | | | | | | | | | | |  |
|  |  | 37°C control | 0.0 | 0.0 | 1.6 | 10.4 | 40.8 | 6.2 | 42.5 | 2.6 | 0.0 | 16.5 | 0.0 | 0.8 | 0.0 | 0.0 | 0.3 | 0.4 | 0.4 | 122.6 |
|  |  | 60°C control | 0.0 | 0.0 | 1.4 | 7.4 | 34.7 | 2.7 | 34.9 | 1.7 | 0.0 | 8.9 | 0.0 | 0.7 | 0.0 | 0.0 | 0.2 | 0.1 | 0.0 | 92.6 |
|  |  | 10% bromelain | 37.7 | 8.0 | 8.5 | 0.0 | 171.9 | 20.7 | 111.3 | 33.7 | 2.8 | 596.3 | 26.9 | 16.6 | 33.2 | 237.1 | 0.0 | 64.3 | 25.1 | 1394.1 |
|  |  | 5% pancreatin | 24.6 | 10.2 | 10.2 | 28.4 | 57.0 | 61.3 | 53.9 | 16.7 | 0.0 | 843.5 | 50.2 | 27.1 | 11.4 | 344.0 | 45.1 | 68.8 | 52.3 | 1704.6 |
|  |  | 5% chymotrypsin | 0.0 | 0.0 | 4.6 | 7.1 | 114.0 | 40.4 | 70.0 | 12.8 | 17.0 | 1187.2 | 30.1 | 3.0 | 8.7 | 72.4 | 18.7 | 30.8 | 6.5 | 1623.5 |
|  |  | 10% papain | 25.7 | 13.9 | 35.1 | 2.3 | 203.8 | 29.2 | 65.2 | 23.8 | 17.5 | 968.6 | 5.9 | 14.5 | 14.4 | 84.5 | 6.4 | 34.2 | 8.6 | 1553.6 |
|  |  |  | **Retentate 2** | | | | | | | | | | | | | | | | |  |
|  |  | 37°C control | 0.0 | 1.4 | 1.7 | 13.8 | 45.4 | 10.6 | 51.3 | 3.1 | 0.0 | 9.7 | 0.5 | 0.2 | 0.0 | 0.7 | 0.3 | 0.4 | 0.0 | 139.1 |
|  |  | 60°C control | 0.0 | 0.0 | 1.8 | 14.3 | 49.8 | 13.3 | 7.6 | 3.3 | 0.9 | 7.9 | 0.6 | 0.7 | 0.0 | 0.7 | 0.0 | 0.0 | 0.3 | 101.2 |
|  |  | 10% bromelain | 4.0 | 11.5 | 9.0 | 0.0 | 161.3 | 47.2 | 122.4 | 34.2 | 7.6 | 323.8 | 181.2 | 11.9 | 26.8 | 79.8 | 2.3 | 24.4 | 15.5 | 1062.9 |
|  |  | 10% pancreatin | 67.1 | 25.3 | 18.4 | 41.6 | 69.8 | 97.9 | 110.9 | 42.9 | 0.0 | 990.7 | 63.3 | 105.7 | 22.2 | 520.6 | 112.7 | 129.1 | 60.2 | 2478.5 |
|  |  | 10% chymotrypsin | 0.0 | 0.0 | 2.6 | 0.0 | 66.9 | 7.6 | 52.3 | 3.6 | 0.0 | 793.9 | 3.8 | 0.0 | 3.1 | 23.9 | 5.7 | 3.6 | 0.5 | 967.4 |
|  |  | 10% papain | 22.8 | 8.7 | 25.5 | 0.0 | 215.1 | 17.7 | 82.9 | 22.3 | 14.6 | 993.6 | 47.5 | 36.8 | 25.5 | 28.8 | 1.2 | 26.0 | 4.2 | 1573.3 |
| **Batch 4** | **20 mL** |  | **Scotta** | | | | | | | | | | | | | | | | |  |
|  |  | 37°C control | 12.8 | 1.8 | 64.8 | 8.5 | 2.9 | 19.8 | 60.6 | 5.2 | 3.0 | 0.1 | 2.6 | 2.2 | 0.7 | 3.9 | 2.7 | 1.2 | 1.2 | 194.1 |
|  |  | 60°C control | 11.7 | 2.5 | 63.7 | 9.2 | 3.3 | 14.3 | 59.2 | 4.9 | 3.2 | 0.0 | 2.9 | 2.3 | 1.4 | 7.9 | 1.9 | 1.7 | 1.9 | 192.0 |
|  |  | 10% bromelain | 17.8 | 26.3 | 33.1 | 12.2 | 8.7 | 10.8 | 58.0 | 11.9 | 2.6 | 21.5 | 23.1 | 3.0 | 5.1 | 27.9 | 5.9 | 15.3 | 9.1 | 292.1 |
|  |  | 10% pancreatin | 110.2 | 123.1 | 323.3 | 56.3 | 70.5 | 134.9 | 87.2 | 64.5 | 12.3 | 64.9 | 93.9 | 54.7 | 15.8 | 183.9 | 41.9 | 98.7 | 56.9 | 1592.9 |
|  |  | 10% chymotrypsin | 11.1 | 2.5 | 65.9 | 7.5 | 1.2 | 8.1 | 40.4 | 34.8 | 3.8 | 29.6 | 19.2 | 6.9 | 16.7 | 23.6 | 19.6 | 6.8 | 4.6 | 302.5 |
|  |  | 10% papain | 18.7 | 19.7 | 71.4 | 35.6 | 8.5 | 20.8 | 39.9 | 13.9 | 8.3 | 252.5 | 29.1 | 12.9 | 11.8 | 98.9 | 15.1 | 26.5 | 15.1 | 698.8 |
|  |  |  | **Retentate 1** | | | | | | | | | | | | | | | | |  |
|  |  | 37°C control | 10.3 | 0.8 | 78.1 | 8.0 | 4.2 | 16.5 | 55.1 | 4.9 | 1.8 | 0.7 | 2.6 | 2.3 | 0.2 | 1.6 | 2.8 | 0.5 | 0.9 | 191.3 |
|  |  | 60°C control | 12.5 | 2.2 | 65.4 | 10.7 | 6.0 | 22.3 | 65.6 | 7.7 | 6.3 | 2.6 | 3.6 | 3.4 | 0.3 | 3.7 | 3.5 | 1.0 | 1.1 | 218.0 |
|  |  | 10% bromelain | 44.3 | 78.3 | 29.4 | 26.2 | 36.9 | 72.0 | 80.5 | 43.6 | 189.1 | 136.4 | 110.8 | 63.8 | 53.1 | 313.0 | 75.8 | 95.8 | 75.1 | 1524.4 |
|  |  | 5% pancreatin | 104.4 | 167.5 | 502.4 | 85.2 | 102.2 | 337.3 | 141.2 | 90.9 | 66.3 | 637.5 | 328.1 | 131.5 | 72.9 | 589.2 | 166.3 | 340.3 | 245.3 | 4108.7 |
|  |  | 5% chymotrypsin | 10.9 | 6.7 | 84.0 | 16.7 | 3.5 | 33.2 | 76.8 | 10.2 | 64.7 | 190.0 | 65.2 | 49.6 | 57.6 | 229.7 | 46.7 | 98.5 | 44.5 | 1088.7 |
|  |  | 10% papain | 52.6 | 68.7 | 146.4 | 102.7 | 13.3 | 37.0 | 80.6 | 33.2 | 112.8 | 726.0 | 93.3 | 59.2 | 43.2 | 403.8 | 63.6 | 98.0 | 66.7 | 2201.2 |
|  |  |  | **Retentate 2** | | | | | | | | | | | | | | | | |  |
|  |  | 37°C control | 27.4 | 1.0 | 140.8 | 16.4 | 5.5 | 36.6 | 144.8 | 5.1 | 2.9 | 3.9 | 1.1 | 2.6 | 1.0 | 4.7 | 1.0 | 1.4 | 2.7 | 398.7 |
|  |  | 60°C control | 24.0 | 1.2 | 124.3 | 6.4 | 5.6 | 33.3 | 125.7 | 3.6 | 2.7 | 5.5 | 1.3 | 2.9 | 0.7 | 5.5 | 1.1 | 1.4 | 5.5 | 350.7 |
|  |  | 10% bromelain | 72.9 | 91.5 | 40.9 | 23.0 | 29.4 | 43.1 | 183.9 | 40.8 | 238.4 | 211.8 | 69.8 | 35.6 | 26.0 | 200.7 | 59.2 | 70.3 | 43.0 | 1480.2 |
|  |  | 10% pancreatin | 245.7 | 288.4 | 774.7 | 122.2 | 173.4 | 274.8 | 304.7 | 162.2 | 66.6 | 268.4 | 260.0 | 201.4 | 64.0 | 492.3 | 154.9 | 293.7 | 160.0 | 4307.4 |
|  |  | 10% chymotrypsin | 17.9 | 4.1 | 58.9 | 5.7 | 1.7 | 14.8 | 80.0 | 71.6 | 12.1 | 145.9 | 21.9 | 14.0 | 23.5 | 55.9 | 23.6 | 26.2 | 14.9 | 592.6 |
|  |  | 10% papain | 86.5 | 82.3 | 263.5 | 114.5 | 24.0 | 50.3 | 227.4 | 46.2 | 170.3 | 734.2 | 92.5 | 89.6 | 35.8 | 317.7 | 51.7 | 59.6 | 47.5 | 2493.6 |
|  | **200 mL** |  | **Scotta** | | | | | | | | | | | | | | | | |  |
|  |  | 37°C control | 0.6 | 0.0 | 2.6 | 0.0 | 46.0 | 33.3 | 37.5 | 6.9 | 4.8 | 34.6 | 2.9 | 2.8 | 0.2 | 4.8 | 0.7 | 0.9 | 0.7 | 179.2 |
|  |  | 60°C control | 1.7 | 9.1 | 1.3 | 7.0 | 64.1 | 47.0 | 74.1 | 12.0 | 4.5 | 52.4 | 3.7 | 3.4 | 0.2 | 12.3 | 1.2 | 2.0 | 1.5 | 297.4 |
|  |  | 10% pancreatin | 102.4 | 120.5 | 340.0 | 83.4 | 73.4 | 134.8 | 120.0 | 73.2 | 34.2 | 66.6 | 112.7 | 63.8 | 22.4 | 192.9 | 50.5 | 115.7 | 66.7 | 1773.4 |
|  |  | 3% pancreatin | 36.1 | 33.5 | 140.6 | 26.6 | 27.6 | 52.0 | 65.8 | 23.4 | 9.9 | 74.4 | 49.3 | 28.2 | 8.6 | 86.5 | 25.2 | 53.1 | 30.2 | 771.0 |
|  |  | 10% papain | 18.1 | 22.9 | 62.1 | 54.3 | 8.5 | 20.6 | 49.7 | 14.2 | 29.3 | 155.6 | 22.9 | 11.7 | 6.2 | 82.8 | 11.7 | 21.2 | 14.0 | 605.7 |
|  |  | 5% papain | 15.5 | 13.1 | 72.0 | 31.2 | 5.2 | 9.7 | 49.8 | 10.0 | 22.1 | 21.0 | 18.5 | 15.2 | 9.4 | 62.4 | 7.3 | 14.6 | 9.6 | 386.6 |
|  |  |  | **Retentate 1** | | | | | | | | | | | | | | | | |  |
|  |  | 37°C control | 0.0 | 0.0 | 1.2 | 7.9 | 28.9 | 6.3 | 31.1 | 1.9 | 1.3 | 33.6 | 1.2 | 0.5 | 0.0 | 1.3 | 0.2 | 0.7 | 0.1 | 116.1 |
|  |  | 60°C control | 0.9 | 1.4 | 1.6 | 5.1 | 16.6 | 4.5 | 19.5 | 1.4 | 1.0 | 5.6 | 0.8 | 0.7 | 0.0 | 1.3 | 0.4 | 0.5 | 0.4 | 61.7 |
|  |  | 5% pancreatin | 112.6 | 125.4 | 381.4 | 114.3 | 87.9 | 312.2 | 177.2 | 79.9 | 89.1 | 574.5 | 361.8 | 122.1 | 67.0 | 509.9 | 157.2 | 323.0 | 234.5 | 3830.0 |
|  |  | 3% pancreatin | 60.4 | 61.8 | 237.0 | 74.8 | 45.2 | 198.1 | 93.3 | 43.4 | 69.3 | 498.9 | 186.1 | 74.5 | 41.8 | 357.2 | 104.4 | 214.9 | 155.0 | 2516.1 |
|  |  | 10% papain | 24.1 | 39.4 | 102.2 | 106.5 | 15.8 | 27.4 | 82.0 | 30.1 | 123.5 | 136.9 | 135.3 | 43.3 | 48.6 | 263.6 | 46.3 | 106.0 | 45.6 | 1376.6 |
|  |  | 5% papain | 36.7 | 25.2 | 115.8 | 87.0 | 12.6 | 17.4 | 95.3 | 24.4 | 161.0 | 631.7 | 154.1 | 41.8 | 88.4 | 165.1 | 65.4 | 53.3 | 51.7 | 1826.9 |
|  |  |  | **Retentate 2** | | | | | | | | | | | | | | | | |  |
|  |  | 37°C control | 1.0 | 2.8 | 3.0 | 12.7 | 41.8 | 8.1 | 50.3 | 3.3 | 1.8 | 9.6 | 1.1 | 1.0 | 0.3 | 3.1 | 0.4 | 0.5 | 0.4 | 141.1 |
|  |  | 60°C control | 0.5 | 0.9 | 0.9 | 5.3 | 15.0 | 4.7 | 22.4 | 0.8 | 0.8 | 2.5 | 0.3 | 0.5 | 0.0 | 0.4 | 0.2 | 0.2 | 0.1 | 55.6 |
|  |  | 10% pancreatin | 335.5 | 391.3 | 1054.4 | 229.3 | 260.4 | 406.6 | 379.1 | 237.6 | 106.2 | 216.8 | 368.8 | 262.4 | 88.4 | 663.8 | 204.9 | 363.7 | 214.7 | 5784.1 |
|  |  | 3% pancreatin | 161.7 | 163.6 | 576.0 | 136.0 | 140.4 | 276.9 | 345.8 | 99.3 | 45.7 | 221.3 | 216.4 | 186.3 | 56.7 | 429.8 | 159.4 | 282.0 | 139.9 | 3637.1 |
|  |  | 10% papain | 74.5 | 73.9 | 198.0 | 115.4 | 19.6 | 34.4 | 180.6 | 36.2 | 117.3 | 542.0 | 66.4 | 73.5 | 51.5 | 285.7 | 40.8 | 49.7 | 41.5 | 2001.1 |
|  |  | 5% papain | 82.7 | 54.6 | 194.6 | 132.4 | 18.5 | 62.3 | 312.6 | 34.6 | 158.4 | 783.7 | 133.9 | 65.4 | 63.5 | 336.4 | 59.3 | 54.0 | 53.2 | 2600.1 |
|  |  |  | **Retentate 1** | | | | | | | | | | | | | | | | |  |
| **Batch 5** | **20 mL** | 37°C control | 51.3 | 29.6 | 137.7 | 230.4 | 19.6 | 99.7 | 398.1 | 66.6 | 63.4 | 103.9 | 61.5 | 35.7 | 29.4 | 106.4 | 812.6 | 35.1 | 42.8 | 2323.9 |
|  |  | 60°C control | 79.5 | 43.0 | 189.1 | 362.2 | 29.8 | 139.5 | 527.3 | 109.8 | 68.7 | 102.8 | 85.5 | 57.2 | 65.7 | 137.9 | 1463.1 | 75.2 | 57.0 | 3593.3 |
|  |  | 3% pancreatin | 161.7 | 176.3 | 576.0 | 281.8 | 131.3 | 597.4 | 319.0 | 146.3 | 371.6 | 10444.2 | 1181.4 | 509.0 | 1450.8 | 1492.5 | 610.8 | 901.3 | 850.6 | 20202.0 |
|  |  | 5% papain | 26.4 | 311.0 | 425.0 | 640.7 | 116.7 | 189.7 | 423.0 | 334.8 | 1291.1 | 7531.5 | 2213.8 | 787.5 | 374.1 | 1394.1 | 481.0 | 349.7 | 374.4 | 17264.4 |
|  | **200 mL** | 37°C control | 39.6 | 18.0 | 173.6 | 194.9 | 27.0 | 114.5 | 338.0 | 59.8 | 49.9 | 63.1 | 49.6 | 30.8 | 33.0 | 91.2 | 675.2 | 34.4 | 36.0 | 2028.7 |
|  |  | 60°C control | 33.0 | 18.5 | 107.9 | 182.9 | 19.2 | 76.4 | 267.7 | 49.9 | 51.1 | 205.6 | 42.9 | 27.7 | 32.0 | 65.6 | 682.1 | 20.2 | 20.2 | 1902.7 |
|  |  | 3% pancreatin | 183.0 | 502.4 | 727.7 | 1048.5 | 235.3 | 1461.3 | 617.7 | 305.5 | 677.4 | 13808.8 | 1736.4 | 1024.5 | 699.3 | 3151.6 | 1449.0 | 2480.0 | 1610.2 | 31718.6 |
|  |  | 5% papain | 97.2 | 421.5 | 333.9 | 772.8 | 94.0 | 265.4 | 398.5 | 394.3 | 1537.4 | 8668.3 | 2661.5 | 801.6 | 569.6 | 1895.0 | 584.9 | 381.7 | 479.1 | 20356.6 |
|  | **2L** | 3% pancreatin | 242.4 | 501.6 | 775.2 | 975.1 | 852.5 | 1423.6 | 432.8 | 325.3 | 1412.0 | 10685.5 | 1900.6 | 1134.2 | 643.5 | 2135.6 | 2191.5 | 2902.4 | 1599.3 | 30133.0 |
|  |  | 5% papain | 103.3 | 159.8 | 191.9 | 618.8 | 51.3 | 141.2 | 234.1 | 148.1 | 873.5 | 5051.5 | 1604.8 | 343.1 | 384.8 | 1012.3 | 297.9 | 240.5 | 363.5 | 11820.4 |
