# Supplementary figures and images for: Enzymatic production of bioactive peptides from scotta, an exhausted by-product of ricotta cheese processing
Source: PLoS One. 2019 Dec 30;14(12):e0226834. doi: 10.1371/journal.pone.0226834 (PMC6936807; doi:10.1371/journal.pone.0226834)

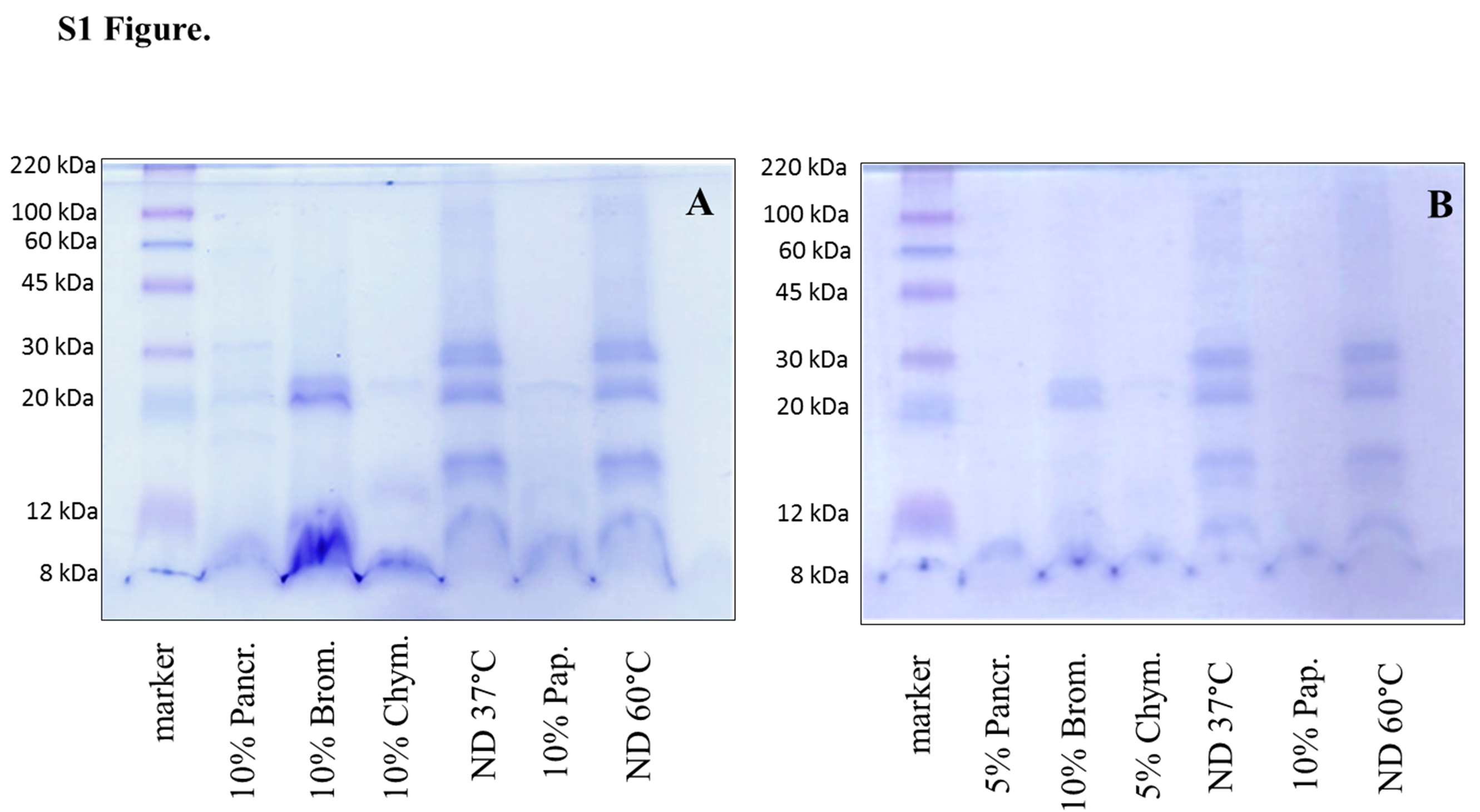

Supplement: S1 Fig — Example of molecular mass distribution (mono-dimensional SDS-PAGE) of initial protein fractions (scotta (A) and retentate 1 (B)) and related peptides obtained after different protease treatments of batch 4 (20 mL incubation volumes). Pancr, pancreatin; Brom, bromelain; Chym, chymotrypsin; Pap, papain; ND, not digested control. (TIF) [file pone.0226834.s003.tif]

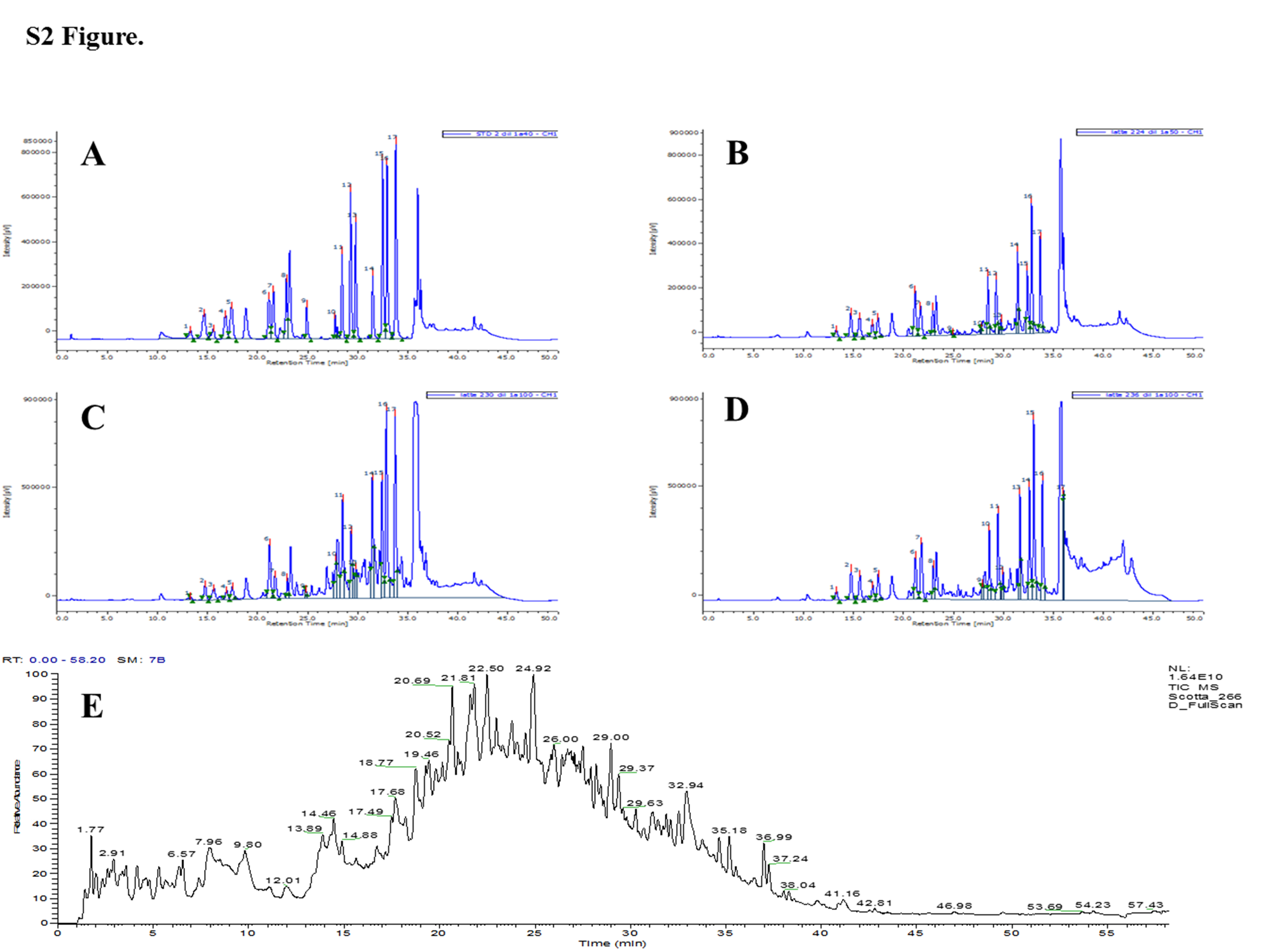

Supplement: S2 Fig — (A-D) HPLC-fluorimeter chromatograms reporting the identification of free amino acids after Pancreatin digestion of initial fractions (20 mL incubation volume). Amino acids standards (A), Scotta (10% Pancreatin, 1h 37°C) (B), Retentate 1 (5% Pancreatin 1 h 37°C) (C), Retentate 2 (10% Pancreatin 1h 37°C) (D). Amino acids: ser (1), asp (2), his (3), glu (4), gly (5), arg (6), thr (7), ala (8), pro (9), cys (10), tyr (11), val (12), met (13), lys (14), ile (15), leu (16), phe (17). (E) Reverse phase UHPLC/ESI-MS chromatogram (Full Scan) of < 5 kDa fraction obtained from retentate 1 sample hydrolysed with 5% Papain 1h at 60°C. (TIF) [file pone.0226834.s004.tif]
